# Autophagy: regulating the seesaw of bone–fat balance

**DOI:** 10.3389/fcell.2025.1465092

**Published:** 2025-02-24

**Authors:** Boya Zhang, Jing Cui, Xu Zhang, Ziyi Pan, Liuyi Du, RongRong Ye, Linlin Wen, Wenhao Zhai, Lei Huang, Daowei Li, Hongchen Sun

**Affiliations:** ^1^ Jilin Provincial Key Laboratory of Tooth Development and Bone Remodeling, School and Hospital of Stomatology, Jilin University, Changchun, China; ^2^ Department of Oral Pathology, Hospital of Stomatology, Jilin University, Changchun, China

**Keywords:** autophagy, metabolism, lipid metabolism, bone metabolism, bone diseases

## Abstract

The interrelationship between bone and fat can be described as a seesaw in bone homeostasis, in which both osteogenesis and adipogenesis occur in a delicate balance. Osteoblasts and adipocytes share a common origin and play key roles in osteogenesis and adipogenesis. Bone–fat balance indicates osteogenesis and adipogenesis keeps a balance for concordant distribution of trabecular bone and bone marrow adipose tissue in bone, thereby leading to the balance between bone metabolism and lipid metabolism. Bone–fat balance is crucial for metabolic health. When disrupted by various factors, this balance can lead to several bone-related metabolic diseases and systemic disorders, such as obesity, osteoporosis, and osteoarthritis. Recent research highlights the role of autophagy dysfunction in these metabolic conditions. Restoring autophagic function can help restore metabolic homeostasis and re-establish the bone–fat balance. The current review explores the factors that regulate bone–fat balance, the consequences of imbalance under pathological conditions, and the potential of autophagy modulation as a therapeutic approach. Overall, it can be concluded that targeting autophagy presents a promising strategy for treating metabolic disorders and restoring bone–fat balance.

## 1 Introduction

A delicate balance exists between a bone and its fat levels. In case of an imbalance, bone dysfunction and lipid metabolic disorders can occur. Factors such as aging, hormonal changes, and energy imbalances can disrupt lipid metabolism, impair adipose tissue function, and increase bone marrow adipose tissue (BMAT) (Chandra et al., 2022; Devlin, 2011; Santopaolo et al., 2020; Zaidi et al., 2018). Obesity is also associated with a high risk of fracture and bone loss (Ali et al., 2022; Rinonapoli et al., 2021). Although BMAT is sometimes considered detrimental to bone mass, it has been shown to provide energy during periods of energy deficiency (Li et al., 2022a; Li et al., 2022b; Karsenty, 2006). Therefore, it becomes crucial to explore the underlying mechanism of bone–fat balance.

Bone–fat balance is the balance between BMAT and bone mass, and it is critical in maintaining bone homeostasis. The bone–fat balance is based on the differentiation of mesenchymal stem cells (MSCs) into either osteoblasts or adipocytes (Chen et al., 2016). Osteogenesis inhibits adipogenesis, while adipogenesis suppresses osteoblast differentiation, thereby creating a competitive relationship that ensures the appropriate distribution of bone and fat in the skeleton (Li et al., 2018a; Yuan et al., 2016). Once the balance is broken, the increasing one is at the expense of the decreasing one, like the seesaw. Disruption of this balance leads to increased BMAT, lipid metabolism disorders, reduced bone mass, and bone dysfunction (Tencerova et al., 2018; Deng et al., 2021).

Autophagy, a cellular process of degrading intracellular substances, maintains a dynamic cycle of energy in the body (Montaseri et al., 2020). A bone requires an enormous amount of energy. Autophagy helps remove metabolic products from the bone and provides the energy necessary to maintain normal bone morphology and function (Wang et al., 2023).

Autophagy also participates in the differentiation, proliferation, and mineralization of osteoblasts (Nollet et al., 2014), and in the bone resorption of osteoclasts (Montaseri et al., 2020). Therefore, autophagy is a regulator between bone and adipose tissue and maintains the bone–fat balance to ensure bone homeostasis. By removing lipid byproducts and reducing fatty acid-induced toxicity, autophagy plays a crucial role in maintaining bone health.

Despite growing research, the exact role of autophagy in regulating bone–fat balance remains poorly understood, particularly under pathological conditions. Addressing this knowledge gap is essential to develop targeted therapies for bone-related metabolic disorders. Earlier studies have discussed the role of autophagy in bone–fat interactions, providing a novel perspective by focusing on how autophagy regulation can restore bone–fat balance and correct metabolic imbalances (Yin et al., 2019). The current study aims to clarify the underlying molecular mechanisms of autophagy in this context and explore its potential as a therapeutic target for different diseases associated with bone metabolism. The present review elaborates on the way how autophagy regulates bone-–fat balance and corrects bone fat imbalance with a view to maintaining bone homeostasis under pathological conditions. It also summarizes the molecular mechanisms of autophagy in bone–fat balance. Overall, understanding the precise mechanisms of autophagy can help us evolve suitable treatment interventions for bone metabolic diseases caused by bone–fat imbalance.

## 2 Autophagy in bone and fat

### 2.1 An outview of autophagy

Autophagy or “self-eating,” is a catabolic process that transports cytosol and cell organelles to the vacuole or lysosome for degradation, macromolecule turnover, and recycling building blocks (Farré et al., 2009; Germain and Kim, 2020). Autophagy can be categorized into macroautophagy, microautophagy, and chaperone-mediated autophagy (Oku and Sakai, 2018). Macroautophagy, the most well-characterized category, is sometimes referred to as “autophagy” (Klionsky, 2005). This process is induced by numerous stress factors, such as starvation, hypoxia, and hormonal changes, thereby helping cells adapt and maintain homeostasis (Wen and Klionsky, 2016).

Autophagy is activated by starvation and the accumulation of metabolic products to decompose stored nutrients and remove excessive metabolic wastes (Galluzzi et al., 2014). The process involves five stages: initiation, vesicle nucleation, vesicle elongation, fusion, and degradation (Klionsky and Emr, 2000). In mammals, autophagy begins with the formation of an autophagosome, a double-membraned vesicle, which engulfs cytosolic components and fuses with the lysosome eventually (Germain and Kim, 2020). The biogenesis of autophagosomes is important and autophagy-related proteins are core mechanisms (Wen and Klionsky, 2016). Overall, autophagy plays a complicated role in the organism to maintain homeostasis.

Although macroautophagy is the most prominent form of autophagy, microautophagy and chaperone-mediated autophagy also perform key functions. Microautophagy directly engulfs cytoplasmic components into the lysosome, whereas chaperone-mediated autophagy selectively transports substrates using chaperone proteins (Tekirdag and Cuervo, 2018). In the current review, we focus on macroautophagy due to its significant role in responding to stress conditions (Klionsky, 2005), which makes it especially relevant for maintaining bone-fat balance and overall bone metabolism. By examining how autophagy responds to stressors such as starvation, hypoxia, and hormonal changes, the present review aims to examine the role of macroautophagy in cellular homeostasis, particularly in bone and lipid metabolism. Autophagy is essential for maintaining the balance between bone and adipose tissue by regulating energy and removing metabolic waste, which is critical for metabolic health.

### 2.2 Autophagy in bone

Skeleton in a majority of vertebrates develops from activated chondrocytes. Chondrocytes proliferate in hypoxic conditions and synthesize extracellular matrix to nourish their terminal differentiation. Chondrocytes possess a highly activated autophagy level in the intervening period for the local hypoxic cartilaginous tissue (Schaaf et al., 2016). Consequently, osteoblast progenitors, osteoclasts, blood vessel endothelial cells, and hematopoietic cells are removed into hypertrophic cartilage from perichondrium (Berendsen and Olsen, 2015), thereby promoting capillary ingrowth and differentiation of osteoblasts (Buck and Dumanian, 2012). The defection of autophagy has been proven to disturb the functions of normal bone cells. In autophagy, metabolites and dysfunctional organelles are eliminated to promote a normal metabolic cycle. Autophagy plays a crucial role in bone formation due to regulated activity and interaction between BMMSCs (bone marrow mesenchymal stem cells), osteoblasts, osteoclasts, and osteocytes to establish a stabilizing environment. Uncontrolled autophagy also serves as a hub of metabolic products in the body and can cause destructive bone homeostasis and metabolic disease.

### 2.3 Autophagy in fat

During starvation, greater autophagy occurs and autophagy is damaged in the process of obesity (Yang et al., 2010). Despite the high expression of autophagy-related genes, autophagic flux declines in adipocytes because of chronic inflammation in adipose tissue (Soussi et al., 2016). However, autophagy serves as a protective mechanism to remove cellular metabolic products and can reduce obesity-induced pressure. Autophagy mediates lipolysis, autophagosomes containing lipid droplets (LDs) combine with lysosomes and reduce accumulation of lipids (Singh et al., 2009). In autophagy, an organism is adversely affected by endoplasmic reticulum (ER) stress (Ozcan et al., 2004). When stress signals facilitate an accumulation of unfolded proteins in the ER, autophagy helps remove these proteins and thus reduces obesity-induced ER stress (Ogata et al., 2006). Furthermore, obesity interferes with the function of mitochondria and increases reactive oxygen species (ROS) and oxidative stress levels in mitochondria. Thus, increasing mitochondrial ROS induces mitophagy and improves lipid metabolism (Wang et al., 2021; He et al., 2021). However, obesity-induced mitophagy may not be a compensatory mechanism because of excessive lipid peroxidation products. It is because mitochondrion, a functional organelle, provides adenosine triphosphates (ATPs) and is severely damaged during mitophagy; this indicates role of mitophagy as a self-rescue measurement in extreme situations.

The above findings elucidate that both bone and adipose tissue contain high levels of metabolism. Autophagy eliminates wastes to keep balance cautiously and protects the organism from damage. However, as a flexible regulator, autophagy has a negative influence under extreme pathological conditions. Hence, autophagy needs to be regulated to keep balance within an appropriate range.

## 3 Bone–fat balance

**FIGURE 1:**
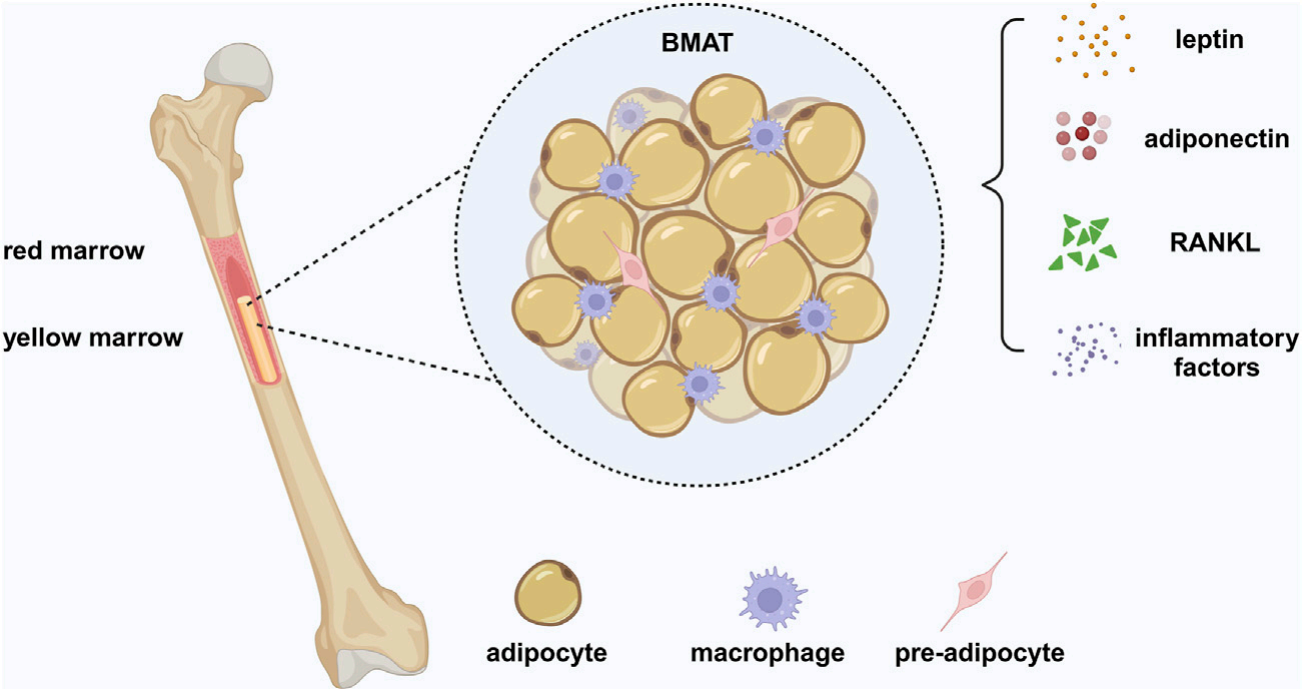
Bone marrow adipose tissue (BMAT). BMAT is a heterogeneous population of cells, containing bone marrow adipocytes (BMADs), preadipocytes, and macrophages. BMAT is a unique form of fat different from white adipose tissue (WAT) and brown adipose tissue (BAT), providing energy to the bone to require the high metabolic demands of bone remodeling and has an endocrine function similar to WAT. BMAT regulates systemic metabolism and bone density by secreting adipokines, such as leptin and adiponectin. BMAD is a significant source of RANKL, which mediates osteoclast-related pathological bone loss by controlling trabecular remodeling. In addition, BMADs and macrophages produce inflammatory factors, leading to a chronic inflammatory environment under pathological conditions. Created in BioRender. Zhang, B. (2025) https://BioRender.com/r61v023.

For many years, bone metabolism and lipid metabolism were considered two unattached fields. Up to now, several studies have revealed a correlation between bone metabolism and lipid metabolism. Discovery of BMAT, a unique adipose tissue located in bone marrow revealed that the metabolic products and secretory cytokines produced by BMAT affect the bone (Figure 1.). Moreover, bone metabolism affects systematic metabolism through the secretion of bone and regulation of BMAT. Therefore, there is a balance between bone and adipose tissue, associated with bone homeostasis and systemic metabolic homeostasis. In a narrow sense, bone-fat balance reveals the balance between osteogenesis and adipogenesis, and results in the equilibrium of trabecular bone and BMAT in the skeleton, which involves the interaction between bone metabolism and lipid metabolism due to from a broad perspective.

Bone-fat balance depends upon the differentiation of mesenchymal stem cells (MSCs). Osteogenesis and adipogenesis compete with each other. MSCs are involved in osteogenesis at the expense of adipogenic formation; adipogenesis inhibits osteoblast differentiation of MSCs (Li et al., 2018a; Yuan et al., 2016). This inverse relationship resembles a seesaw, when one process is promoted, the other is inhibited, and *vice versa*. The differentiation of MSCs toward specific lineages is regulated by different cellular signaling pathways and multiple transcription factors. Signaling pathways involved in the differentiation of MSCs play a role in both osteogenesis and adipogenesis rather than applying differentiation separately. The main signaling pathway during osteogenesis is the Wnt/β-catenin pathway, which mediates an intercellular signaling network that strictly regulates adipose tissue expansion, inhibits adipocyte differentiation by suppressing peroxisome proliferator-activated receptor-γ (PPAR-γ) and C/EBPα expression, and promotes MSC differentiation into osteoblasts (Christodoulides et al., 2009). Conversely, PPARγ inhibits the Wnt/β-catenin pathway, which is involved in DNA methylation and histone acetylation of C/EBP (Zhao et al., 2013; Takada et al., 2009). PPAR-γ decreases RUNX2 expression in osteoblasts to inhibit osteoblast differentiation and diverts the differentiation pathway toward adipogenesis (Liu et al., 2010; Wan, 2010). PPARγ haploinsufficiency stimulates bone marrow progenitor cells to generate osteoblasts resulting in increased bone mass without affecting differentiated osteogenic lineage cells (Akune et al., 2004). The competition in MSC differentiation creates a delicate equilibrium of bone and fat, resulting in the harmonious distribution of trabecular bone and BMAT in the skeleton. Once this equilibrium gets broken, the increasing bone mass is at the expense of the decreasing one. For example, osteoporosis shows high BMAT components and loose trabecular bone; however, high bone mass (HBM) shows exceeding dense trabecular bone and inhibited adipogenesis (Qiu et al., 2007). Despite the competitive relationship between osteogenesis and adipogenesis, bone and fat restrict and impact each other to maintain the bone-fat balance.

### 3.1 Factors required to maintain bone–fat balance

#### 3.1.1 Adipokines

**FIGURE 2:**
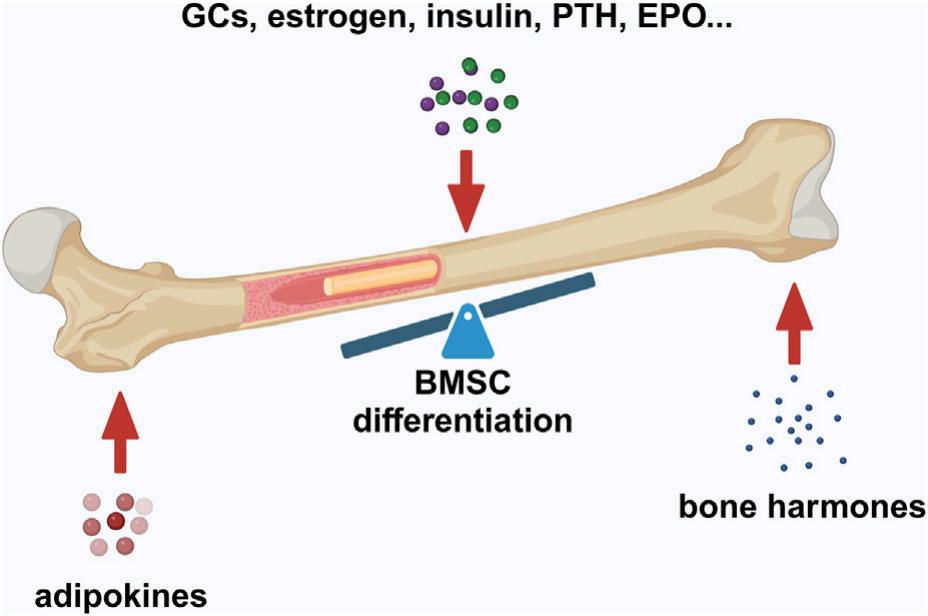
Factors maintaining bone–fat balance. Bone–fat balance is based on MSC differentiation. Additionally, bone–fat balance is maintained by adipokines secreted by BMAT, bone-derived hormones, and other hormones such as GCs, estrogen, insulin, PTH, and EPO. Created in BioRender. Zhang, B. (2025) https://BioRender.com/o20m134.

Adipokines maintain bone homeostasis by engaging bone remodeling and can lead to pathological bone disease, as indicated by skeletal fat infiltration during bone aging (Figure 2) (Ambrosi et al., 2017). Leptin hormone, which is secreted by adipocytes, is positively correlated with fat mass. Leptin has been shown to enhance osteogenesis and inhibit adipogenesis of human MSCs *in vitro* (Thomas et al., 1999). Adiponectin hormone, that is secreted by adipose tissue, can also directly affect bone volume *in vivo* by stimulating osteogenesis and inhibiting osteoclastogenesis (Madel et al., 2021). Obesity induces the expression of pro-inflammatory factors in the bone marrow microenvironment, and association analysis reveals a negative correlation between markers causing inflammation and bone metabolism (Labouesse et al., 2014; Fuggle et al., 2018; Russell et al., 2010).

#### 3.1.2 Bone-derived hormones

A bone can secrete various hormones also to control energy homeostasis and mineral homeostasis as an endocrine organ. Osteocalcin (OCN), secreted by osteoblasts, enhances glucose transport in adipocytes, inhibits the secretion of pro-inflammatory factors, and mediates the release of adiponectin, thereby causing positive effect on bone (Hill et al., 2014). Osteopontins (OPNs), extracellular matrix proteins, are expressed in several tissues and organs. OPN-deficient MSCs tend to differentiate into adipocytes with impaired capacity for bone formation and increased fat deposition *in vivo*. The possible mechanism behind this is that OPNs inhibit C/EBPα signaling, thereby suppressing adipogenesis (Chen et al., 2014). Bone morphogenetic protein (BMP) plays a dual role in bone and adipose tissue. BMP2, an approved therapeutic agent for bone regeneration, has shown excellent ability to promote bone healing at fracture sites in high-fat diet (HFD)-fed mice; however, BMP2 also promotes adipogenesis (Bhatti et al., 2021).

#### 3.1.3 Other hormones

Glucocorticoids (GCs) can control bone remodeling as a hormonal factor and also most likely affect bone-fat balance through crosstalk with signaling network, and deletion of GC receptors in bone promotes increased BMAT and decreased bone mass (Sharma et al., 2019). Estrogen inhibits transdifferentiation of osteoblasts into bone marrow adipocytes (BMADs) to maintain the bone-fat balance (Gao et al., 2014). In the absence of estrogenic regulation, the β-linked protein forms a new complex with Estrogen Receptor α (Erα) to induce the accumulation of lipid droplets in osteoblasts, thereby indicating the transdifferentiation of osteoblasts to adipocytes (Foo et al., 2007). Insulin, the most important regulator in glucose metabolism, maintains bone-fat balance. Human adipose-derived stem cells derived from obesity show resistance to insulin, which causes increased adipogenesis and reduced osteogenesis (Rawal et al., 2020). Parathyroid hormone (PTH) participates in bone metabolism by regulating serum calcium and controls bone-fat balance by impacting MSC differentiation and osteoclastogenesis via the RANK-RANKL-OPG pathway (Fan et al., 2017; Casado-Diaz et al., 2010). BMAT occupies space inside bone marrow during aging-related bone loss and hematopoietic bone marrow loss. Erythropoietin (EPO) maintains bone-fat balance by regulating the hematopoietic ability of bone marrow to prevent defective hematopoietic ability caused by expanding BMAT (Suresh et al., 2020; Noguchi, 2020).

### 3.2 Pathological condition: the imbalance of the seesaw

#### 3.2.1 High bone mass phenotype

Mutation of Wnt pathways-related genes can cause high bone mass (HBM), which is characterized by high trabecular bone density, activated osteogenesis, and reduced adipogenesis (Qiu et al., 2007). Due to the special bone biological property of HBM, the genetic analysis contributes to the converse bone metabolic disease, osteoporosis. Low-density lipoprotein receptor-related protein 5 (LRP5) expresses highly in bone remodeling position (Little et al., 2002), bonding with sclerostin (SOST) to regulate the Wnt signaling pathway negatively. Furthermore, the knockdown of SOST in mice shows a phenotype similar to HBM (Yee et al., 2018). Due to the inhibitory role of the Wnt signaling pathway in adipogenesis, SOST is an important regulator of lipid metabolism (Delgado-Calle and Bellido, 2017). SOST can increase bone marrow adipose tissue formation by inhibiting Wnt signaling in adipocyte progenitor cells (Fairfield et al., 2018). Current research on sclerostin remains limited to the local skeleton and skeletal microenvironment. Still, SOST can be considered a novel target that can connect the skeleton to fat.

#### 3.2.2 Oxidative stress

Oxidative stress indicates that greater levels of oxidants destroy the redox balance, and ROS is widely discussed as a common oxidant (Sies, 2015). ROS affects cells in a dose-dependent way. Low-dose ROS is advantageous to cells; however, high-dose ROS damages cellular viability, proliferation, and functions. ROS accumulates in osteoblasts during osteogenic differentiation, which is related to the activated redox level in the bone remodeling position (Domazetovic et al., 2017). Slight ROS level promotes mineralization but excessive ROS inhibits osteogenesis and induces adipogenesis (Nicolaije et al., 2012; Geissler et al., 2013). Supplement of antioxidants reduces oxidative DNA damage and promotes the proliferation of MSCs (Alves et al., 2013). Upregulation of heme oxygenase-1(HO-1) ), a cellular antioxidant, in MSC-derived adipocytes inhibits adipogenesis and activates the Wnt/β-catenin pathway (Vanella et al., 2013).

#### 3.2.3 Aging

Aging, an important factor, disrupts the bone-fat balance. Aging skeleton shows a higher oxidative stress level, decreasing osteogenesis, increasing BMAT (Chandra et al., 2022; Jilka et al., 2010), and declining sex hormones with aging accelerate bone loss (Almeida et al., 2007). Oxidative stress caused by aging regulates the shift toward adipogenic differentiation (Kim et al., 2012). Higher levels of BMAT in the elderly are negatively associated with low bone mineral density compared to the young due to preferential differentiation of MSC to adipocytes with aging (Shen et al., 2012). Similarly, elevated BMAT-related genes and changing lipid metabolites were observed in aging skeletons (Chandra et al., 2022; Yu et al., 2022). Longitudinal assessment of cellular senescence revealed that BMAT is identified as an essential factor for MSC adipogenicity during bone aging, and inhibition of adipokines could change the fate of MSCs (Chandra et al., 2022). The inverse relationship between increasing bone fat and decreasing bone mass during aging was demonstrated first in spontaneously osteoporotic mice and aged osteoporotic patients (Justesen et al., 2001; Takahashi et al., 1994), which may be related to the lineage tilt of MSCs during aging. Consequently, aging is the primary factor causing an imbalance of MSC differentiation; the aging-related oxidative stress, and reducing sex hormones boost the imbalance.

#### 3.2.4 Inflammation

Adipose tissue is a pro-inflammatory environment. Obese individuals express increasing pro-inflammatory factors and infiltrating Adipose Tissue Macrophages (ATMs) in adipose tissue (Iyengar et al., 2016). An expanding BMAT exists in osteoporosis, and is associated with low bone mineral density and increased fracture risk (Li and Schwartz, 2020; Woods et al., 2020). Although no firm relationship between obesity and osteoporosis has yet been established, a chronic inflammatory environment is suggested to induce osteoporosis in obesity. Lipid metabolism disorders cause a pro-inflammatory environment and induce osteoarthritis. Adipokines released from adipose tissue and metabolites such as fatty acids affect chondrocytes to exhibit a pro-inflammatory phenotype. Obese individuals have shown raised leptin and adiponectin levels compared with healthy controls, and these adipokines act as pro-inflammatory mediators involved in the occurrence and development of osteoarthritis (Vuolteenaho et al., 2012; Ilia et al., 2022; Kroon et al., 2019). Adipokines contribute to inflammation in synovium, production of matrix metalloproteinase (MMP), cartilage degeneration, and bone remodeling in osteoarthritis (OA) (Kroon et al., 2019). Together, these studies indicate a complex role of inflammation in bone-fat balance.

## 4 How autophagy regulates the bone–fat balance

### 4.1 Autophagy determines BMMSC differentiation for the bone–fat balance

**FIGURE 3:**
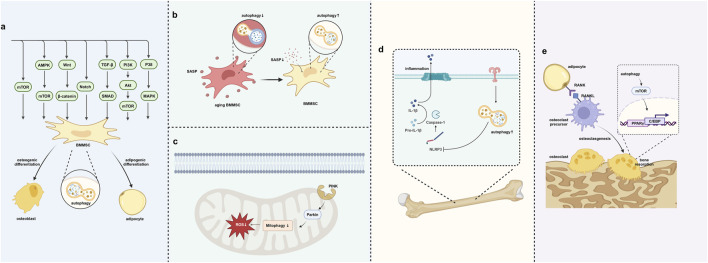
Autophagy regulates the bone–fat balance. **(A)** Autophagy regulates BMMSC differentiation through signaling pathways. **(B)** Autophagy removes SASP to prevent cell aging. **(C)** Autophagy in mitochondria removes oxidative stress in BMMSCs. **(D)** Autophagy helps in removing inflammation within the bone–fat balance context. **(E)** Autophagy’s role in regulating osteoclast differentiation and bone resorption. Created in BioRender. Zhang, B. (2025) https://BioRender.com/l10y832.

BMMSCs can differentiate into various cell types, including osteoblasts, adipocytes, and chondrocytes. The differentiation process is tightly controlled by autophagy, which plays a key role in BMMSC differentiation through multiple signaling pathways. Autophagy maintains cellular homeostasis by eliminating metabolic waste, damaged organelles and proteins, while also determining the differentiation lineage of BMMSC through signaling pathways (Table 1). By regulating these pathways, autophagy helps maintain bone-fat balance, supports healthy bone metabolism, and mitigates the onset of bone metabolic diseases, such as osteoporosis (Figure 3).

**TABLE 1:** Role of autophagy in regulating BMMSC differentiation through various signaling pathways.

Signaling pathway	Function	Role of autophagy	Impact on BMMSC differentiation
mTOR pathway	Key regulator of cell growth and metabolism, sensing nutrient, and energy status	Autophagy inhibits mTOR activity, promoting bone and fat balance	mTORC1 activation promotes osteogenesis; mTORC2 inhibits adipogenesis
AMPK pathway	Senses cellular energy status and activates under low-energy conditions, thus promoting autophagy while inhibiting mTOR	Autophagy activated by AMPK suppresses adipogenesis and supports osteogenesis	Promotes osteogenesis in BMMSCs while inhibiting adipogenesis
Wnt/β-catenin pathway	Regulates cell fate by controlling β-catenin stability	Autophagy modulates β-catenin degradation or stabilization to maintain Wnt signaling	Promotes osteogenesis by increasing expression of osteogenic genes
Notch pathway	Plays a critical role in cell differentiation, proliferation, and apoptosis	Autophagy affects Notch receptor activity	Activates osteogenesis while inhibiting adipogenesis in BMMSCs
TGF-β/Smad pathway	Regulates cell growth, differentiation, and apoptosis	Autophagy enhances TGF-β signaling, upregulating osteogenesis-related gene expression	Promotes osteogenesis by enhancing osteogenic genes
PI3K/Akt pathway	Regulates cell proliferation, metabolism, and growth, affecting BMMSC differentiation	Autophagy inhibits overactive PI3K/Akt signaling, maintaining osteogenic capacity	PI3K/Akt activation promotes adipogenesis
p38 MAPK pathway	Plays a key role in stress responses and differentiation	Autophagy modulates p38 MAPK signaling to maintain proper BMMSC differentiation	p38 MAPK activation favors osteogenesis and inhibits adipogenesis. Autophagy regulates this balance

#### 4.1.1 mTOR signaling pathway

The interplay between mTOR signaling and autophagy is essential for maintaining bone-fat balance. mTOR signaling occurs through two complexes: mTORC1 (mTOR complex 1) and mTORC2 (mTOR complex 2) (Yang and Guan, 2007). mTORC1 is associated with osteogenesis in BMMSCs, while mTORC2 helps inhibit adipogenesis, thereby maintaining bone-fat balance (Martin et al., 2015). Activation of mTORC1 promotes osteogenesis, while its excessive activation leads to adipogenesis and the accumulation of BMAT, which has been associated with bone loss (Choi et al., 2018). Increased adipogenesis and reduced osteogenesis contribute to age-related bone diseases such as osteoporosis. Autophagy serves as a protective mechanism by degrading fatty acids and cellular debris, thus maintaining bone microenvironment. Modulating autophagy and mTORC1 activity may offer several potential strategies to counteract or slow these age-related changes in BMMSC differentiation.

#### 4.1.2 AMPK signaling pathway

AMPK (5′AMP-activated protein kinase) is a cellular energy sensor activated under conditions of low energy. It regulates cell growth, metabolism, and autophagy by inhibiting the mTOR pathway (Kim et al., 2011). AMPK supports BMMSC differentiation toward osteogenesis by activating autophagy (Wang et al., 2016). AMPK-activated autophagy has been suggested to increase the expression of bone formation-related genes (Runx2 and osteocalcin) (Pantovic et al., 2013). Simultaneously, AMPK inhibits the expression of adipogenesis-related genes (PPARγ and C/EBPα) through autophagy, thereby reducing the differentiation of BMMSCs into adipogenesis (Chava et al., 2018).

#### 4.1.3 Wnt signaling pathway

The Wnt signaling pathway is a crucial regulator in regulating BMMSC differentiation, particularly during osteogenesis. Wnt signals regulate the differentiation of BMMSC toward osteogenesis by stabilizing or activating β-catenin. Autophagy facilitates BMMSC osteogenesis by modulating β-catenin activity and enhances the Wnt/β-catenin signaling transmission (Ge et al., 2023). Autophagy activation helps remove inhibitory factors that block Wnt signaling, thereby increasing the nuclear translocation of β-catenin and the expression of genes related to osteogenesis (Li et al., 2019). Upregulation of β-catenin increases the expression of osteogenesis-related transcription factors, thereby promoting the osteogenesis process (Chen et al., 2007). In BMMSCs, β-catenin activation is closely associated with adipogenesis inhibition (Luo et al., 2019), suggesting the role of autophagy in reducing adipogenesis and promoting osteogenesis by activating Wnt/β-Catenin signaling.

#### 4.1.4 Notch signaling pathway

Notch signaling is a critical intercellular pathway that regulates various biological processes, such as cell differentiation, proliferation, and fate determination. Autophagy affects the differentiation of MSCs in different directions by regulating the degradation and stability of Notch receptors and altering their activity (Rao et al., 2021; Qiu et al., 2017). Activation of Notch signaling upregulates the expression of bone formation-related transcription factors, thereby promoting osteogenesis (Tezuka et al., 2002; Xu et al., 2018). Notch signaling is also closely related to adipogenesis (Garcés et al., 1997). Autophagy inhibits the expression of adipogenesis-related genes by activating Notch signaling, thus reducing adipocyte differentiation, and maintaining bone-fat balance (Song et al., 2015).

#### 4.1.5 TGF-β/Smad signaling pathway

The TGF-β (transforming growth factor β) signaling pathway is a key regulator of cell proliferation, differentiation, migration, and survival. In BMMSCs, the TGF-β/Smad signaling pathway primarily regulates their differentiation into osteoblasts, cartilage, and adipocytes. TGF-β activates Smad2/3 to promote bone formation (Finnson et al., 2008; Dong et al., 2020). Conversely, TGF-β can also inhibit adipogenesis through Smad2/3 (Li et al., 2022c). Furthermore, autophagy could affect the transmission of TGF-β/Smad signaling pathway by regulating the activity and degradation mechanism of TGF-β receptors (Stavropoulos et al., 2022; Pokharel et al., 2016; Tong et al., 2019). Autophagy promotes osteogenic differentiation of BMMSCs by removing factors related to the TGF-β/Smad signaling pathway (such as Smad7) and enhancing the activity of the pathway (Li et al., 2020).

#### 4.1.6 PI3K/Akt signaling pathway

The PI3K/Akt signaling pathway plays an important role in the differentiation of BMMSCs. Activation of the PI3K/Akt signaling pathway can inhibit the expression of adipogenesis-related factors (PPAR-γ and C/EBPα), reduce the differentiation of adipocytes, and thus reduce the accumulation of fat in the bone marrow (Hinoi et al., 2014; Song et al., 2017). Autophagy promotes the differentiation of BMMSCs into osteoblasts by enhancing the activity of the PI3K/Akt signaling pathway, thus removing accumulated waste fat (Shi et al., 2025; Feng et al., 2024; Zheng et al., 2023a; Xu et al., 2025a), and maintaining the bone-fat balance. This process is vital for preserving bone metabolism stability and preventing bone-related diseases, such as osteoporosis.

#### 4.1.7 p38 MAPK signaling pathway

Autophagy plays a complex role in regulating the differentiation of BMMSCs through the p38 MAPK signaling pathway. p38 MAPK (mitogen-activated protein kinase), an important kinase, is involved in cellular responses to oxidative stress, inflammation, and other environmental stimuli (Zarubin and Han, 2005). Autophagy modulates p38 MAPK activity by removing oxidative stress and damaged cellular components (Corcelle et al., 2007), thereby promoting osteogenic differentiation of BMMSCs (Su et al., 2024). Additionally, the p38 MAPK signaling pathway inhibits adipogenesis during BMMSC differentiation. Autophagy further supports this inhibition by eliminating excess adipogenic factors, such as PPARγ and C/EBPα, and inhibiting adipocyte differentiation through interactions with the p38 MAPK pathway (Rahman and Kim, 2020; Wang et al., 2022a).

### 4.2 Autophagy manipulates cell fate during aging for the bone–fat balance

Senescent cells exhibit reduced autophagic flux (Revuelta and Matheu, 2017). A downregulated autophagy expression in senescent cells has been suggested to help prevent the accumulation of inflammasome components and damaged mitochondria, both of which could promote chronic activation of pro-inflammatory signals (Stahl et al., 2018). In a bone, decreased autophagic flux due to cellular senescence impairs bone regeneration and leads to BMAT expansion (Yu et al., 2018; Salazar et al., 2020). Therefore, enhancing autophagic activity to prolong MSC lifespan offers a promising strategy to improve bone-fat balance (Salminen and Kaarniranta, 2009).

#### 4.2.1 Removal of senescence-associated factors

Senescence-associated secretory phenotype (SASP) factors include pro-inflammatory cytokines (e.g., IL-1, IL-6, TNF-α) and matrix-degrading enzymes that contribute to chronic inflammation and impair the bone microenvironment (Young and Narita, 2009). Autophagy breaks down these components, thus reducing their pro-adipogenic and anti-osteogenic effects (Bai et al., 2023; Yang et al., 2023; Ejaz et al., 2016). Autophagy limits the inflammatory signals that promote bone marrow adiposity and bone loss by removing SASP. Autophagy facilitates the degradation of SASP components, such as pro-inflammatory cytokines (e.g., IL-1β (Guo and Wu, 2021), IL-6 (Zheng et al., 2023b), TNF-α (Zheng et al., 2021)) and MMPs, which disrupt the bone-fat balance by enhancing adipogenesis and inhibiting osteogenesis.

Autophagy helps regulate extracellular matrix (ECM) composition by removing senescence-related MMPs that degrade bone matrix and promote fat deposition (Li et al., 2018b). This regulation preserves the structural integrity of a bone and limits marrow fat expansion (Wood et al., 2020; Zhang et al., 2018a; Jiang et al., 2016). Senescent cells upregulate MMPs, as part of the SASP. These MMPs degrade key components of the bone matrix, such as collagen and proteoglycans, leading to impaired bone formation and structural weakening (Xu et al., 2025b). Excessive MMP activity also promotes fat accumulation in the bone marrow by disrupting the niche required for osteogenesis (Zhang et al., 2018a; Allegra et al., 2018). Autophagy-mediated removal of MMPs prevents excessive degradation of collagen and other ECM components, supporting bone matrix integrity (Lu et al., 2021; Huang et al., 2017).

#### 4.2.2 Selective autophagy receptors

Selective autophagy enables cells to target and degrade specific senescence-associated molecules (Kirkin and Rogov, 2019). This targeted degradation is essential for maintaining bone-fat balance by promoting osteogenesis and limiting adipogenesis. Key receptors such as optineurin (OPTN) and p62 play pivotal roles in this process. Receptors like OPTN mediate selective autophagy, removing aging-related proteins such as fatty acid-binding proteins (FABPs). This process enhances osteogenic differentiation and reduces fat accumulation in bone marrow (Liu et al., 2021; Xu et al., 2024). p62 binds to ubiquitinated senescence-associated proteins and directs them to autophagosomes for degradation (Salazar et al., 2020; Zhang et al., 2025a). This process mitigates the accumulation of pro-inflammatory SASP components, thereby disrupting bone microenvironment (Hu et al., 2023; Chen et al., 2022a). p62-mediated autophagy reduces inflammation, curtails adipogenesis, and enhances osteogenesis (Zhang et al., 2025b; Agas et al., 2022; Chen et al., 2021; Lacava et al., 2019).

#### 4.2.3 Regulation of p53 and SIRT1 pathways

Autophagy modulates senescence-related pathways, including p53 and SIRT1.

p53 is a key regulator of cellular senescence, and its activation inhibits osteogenesis while promoting adipogenesis. Autophagy helps regulate p53 activity, restoring the bone-fat balance. Additionally, Autophagy regulates the levels of ROS and p53 to control the biological properties of MSCs; this is corroborated by previous findings that show that inhibition of autophagy reduces osteogenic differentiation and proliferation while increasing adipogenesis in young MSCs (Ma et al., 2018). Activation of p53 in senescent MSCs impairs osteogenic differentiation, as shown by incomplete upregulation of the transcription factor Osterix (Despars et al., 2013). P53 inhibits osteogenesis by forming the complex with E3 ligase Murine double minute 2 (Mdm2), which also promotes lipogenic differentiation by promoting C/EBP δ expression. Disruption of this complex using Mdm2 inhibitors enhances osteogenic differentiation and highlights the regulatory role of autophagy in MSC differentiation (Hallenborg et al., 2012) (Daniele et al., 2019).

SIRT1 promotes osteogenesis by deacetylating transcription factors FOXO3 while inhibiting adipogenesis. Autophagy stabilizes SIRT1 levels, enhancing its protective role against senescence-induced imbalance. SIRT1 (Sir2), an NAD^+^-dependent deacetylase, is degraded through the autophagy pathway during aging (Xu et al., 2020). SIRT1 upregulation increases adipogenesis and reduces osteogenesis in female mice with chronic energy deficiency (Louvet et al., 2020). This gender-specific effect is possibly related to the crosstalk between SIRT1 and Erα, which upregulates SIRT1 expression and inhibits autophagy and adipogenesis. Moreover, SIRT1 induces deacetylation of Erα (Tao et al., 2021). These findings suggest that SIRT1 regulates autophagy in a gender-specific way in autophagy and adipogenesis, potentially contributing to bone mass and body weight imbalances as observed in postmenopausal osteoporosis. FOXO3 is an essential molecule that assists SIRT1, deacetylates FOXO3 to attenuate FOXO3-induced apoptosis and promote cell survival, thereby prolonging cell longevity (Brunet et al., 2004). FOXO3, a ROS-sensitive molecule, gets highly acetylated under oxidative stress. SIRT1-mediated deacetylation of FOXO3 activates RUNX2, which is vital for MSC differentiation toward osteogenesis (Lin et al., 2018). Targeting FOXO3 with miRNAs to upregulate its expression enhances autophagy and promotes osteogenic differentiation in MSCs (Long et al., 2021).

### 4.3 Autophagy removes oxidative stress for the bone–fat balance

Autophagy, a cellular process that involves the degradation of damaged organelles and misfolded proteins, plays a crucial role in managing oxidative stress. By removing damaged mitochondria (mitophagy) and other reactive molecules, autophagy helps protect cells from oxidative damage and thus promotes cellular homeostasis (Shu et al., 2021; Shen et al., 2016). Autophagy is essential for controlling ROS levels, maintaining bone cell function, and regulating fat accumulation in the bone marrow. High ROS levels impair osteogenic differentiation of BMMSCs, thus reducing bone formation (Tan et al., 2015). ROS promotes adipogenic differentiation of BMMSCs, thereby contributing to marrow fat accumulation (Ikeda et al., 2023).

#### 4.3.1 Mitophagy

Autophagy selectively removes damaged mitochondria from a cell, reducing oxidative stress in BMMSCs. Mitophagy removes dysfunctional mitochondria, maintaining a healthy mitochondrial network essential for osteogenesis (Suh et al., 2023). Mitophagy ensures efficient mitochondrial function, supporting ATP production required for bone matrix deposition and osteogenic activity (Fan et al., 2019; Wang et al., 2024a). This process restores cellular homeostasis, by promoting osteogenesis and inhibiting adipogenesis (Feng et al., 2021). Healthy mitochondria are critical for osteoblast differentiation; reduced ROS levels prevent oxidative damage to transcription factors such as Runx2, which are essential for bone formation (Shen et al., 2024). Mitophagy inhibits adipogenic differentiation by decreasing mitochondrial ROS and inhibiting pro-adipogenic factors like PPAR-γ (Wu et al., 2019; Sagar et al., 2023). Efficient mitochondrial turnover shifts BMMSC differentiation away from fat accumulation (Nandy et al., 2023; Liu et al., 2024). This process is regulated by key proteins like PINK1 and Parkin, which tag damaged mitochondria for degradation. This pathway is the primary regulator of mitophagy, supporting osteogenic differentiation while reducing fat deposition (Wang et al., 2024b).

#### 4.3.2 Selective removal of oxidized lipids

Autophagy eliminates oxidized cellular components that interfere with normal signaling pathways and preserves the osteogenic potential of BMMSCs. When the bone-lipid balance is in favor of the fat, lipid metabolism disorder results in lipid peroxidation, which induces oxidative stress to decrease osteogenesis and increase adipogenesis (Almeida et al., 2009). However, autophagy can reduce the stress of lipotoxicity on cells through the management of lipid metabolism. High levels of autophagy were observed in Palmitic Acid (PA)-induced osteoblasts and could be inhibited by the PI3K inhibitor 3-MA, thus protecting osteoblasts from lipotoxicity and cell death (Gunaratnam et al., 2014). Although excessive lipid metabolic products cause oxidative stress, some advantageous fatty acids mitigate damage due to lipid peroxidation. Eicosapentaenoic Acid (EPA), an n-3 PUFA mainly present in fish or fish oil, induces autophagy in various cells to reduce lipotoxicity (Hsu et al., 2018; Yamamoto et al., 2021). EPA induces autophagy and maintains proliferative capacity through the GPR120-mediated AMPK-mTOR signaling pathway in mice MSCs (Gao et al., 2016). Increasing EPA in the diet of mice can reduce inflammation and bone resorption to prevent bone degeneration, possibly through mitochondrial induction of the corresponding oxidative system and autophagy (Gao et al., 2016; Bullon et al., 2013).

### 4.4 Autophagy removes inflammation for the bone–fat balance

Inflammation regulates bone homeostasis in several complex ways. Inflammatory factors stimulate bone healing after bone fracture while promoting osteoclastogenesis and bone resorption. However, Lipopolysaccharide (LPS) during infection and metabolic products secreted by adipose tissue destroy bone-fat balance and cause bone metabolic disease.

LPS released by bacteria mediates inflammatory bone loss due to the infection in periodontitis and osteomyelitis. LPS facilitates bone resorption to regulate osteogenesis negatively and induce adipogenesis. LPS binding protein increases the resistance of adipocytes to inflammation (Moreno-Navarrete et al., 2015). In addition, LPS stimulates NLRP3 to inhibit osteogenesis and enhance adipogenesis (Wang et al., 2017a). Deficiency of NLRP3 in mice reduces bone resorption and inhibits activated inflammatory mediators produced by adipose tissue (Wang et al., 2017a; Alippe et al., 2021; Wu et al., 2023; Vandanmagsar et al., 2011). These studies suggest that NLRP3 is the regulator binding bone-fat balance in inflammation-related bone loss (Behera et al., 2022). Autophagy controls the secretion and degradation of IL-1β and the resulting formation of NLRP3 to alleviate inflammation (Harris et al., 2011). Therefore, inhibition of NLRP3 to upregulate autophagy is speculated to reverse bone-fat balance and revert to homeostasis. Osteoblasts show low autophagy activity, inhibited osteogenic genes, increased pro-inflammatory cytokines, and NLRP3 under inflammation, stimulating autophagy in BMMSCs promotes osteogenesis (Dai et al., 2021; Guo and Wu, 2021). Recently, inhibition of NLRP3 has been shown to upregulate autophagy and decelerate aging of the ovary in mice, which is similar to the mechanism in postmenopausal osteoporosis to rescue bone loss (Navarro-Pando et al., 2021). MCC950, the specific inhibitor of NLRP3, performed an excellent anti-aging feature by increasing the autophagy activity in old mice (Marín-Aguilar et al., 2020).

Although autophagy protects an organism from inflammation, the regulation of autophagy depends on inflammatory signals. Autophagy is the negative regulator of the Wnt signaling pathway. LPS induces autophagy to downregulate the Wnt pathway and osteoclastogenesis to mediate bone loss in periodontitis (Chen et al., 2020a), and acts as an inhibitor of autophagy to reduce bone loss (He et al., 2020). These studies indicate a flexible adaptive capability of autophagy. Autophagy can be a “defender” to remove damage in temperate inflammatory stimulation; however, autophagy is the accomplice under intensive inflammation. There is a need to study thoroughly the adaptation of autophagy.

In addition, autophagy can clear the inflammatory signals due to lipid metabolism disorders. Autophagy upregulates in obesity responding to the secreted pro-inflammatory adipokines (Jansen et al., 2012). OA tends to be caused by high lipid-induced inflammation, so it can be prevented and treated by regulating autophagy. Decreasing the n-6: n-3 Polyunsaturated Fatty Acid (PUFA) ratio in the diet can prevent osteoarthritis, and reducing the n-6: n-3 ratio through fat-1 transgene expression can prevent OA in cartilage and synovium (Wu et al., 2015). Study groups fed with a high n-3 diet (n-6: n-3 ratio of 1.5:1) showed less calcified cartilage damage and reduced calcified cartilage compared with controls provided with a typical western diet (n-6: n-3 ratio of 22:1); it was evaluated by reducing mineralization markers, collagen lysyl hydroxylation, L-Pyr crosslinking, serum pro-inflammatory cytokines and increasing anti-inflammatory cytokines (Knott et al., 2011; Kimmerling et al., 2020). This alteration of the n-6: n-3 PUFA ratio may prevent OA by promoting autophagy in chondrocytes. Both exogenous and endogenous n-3 PUFAs in fat-1 transgenic (TG) mice promote chondrocyte autophagy by downregulating mTORC1 activity for their survival (Huang et al., 2014).

### 4.5 Autophagy aims at osteoclasts for maintaining bone–fat balance

Osteoclasts, along with osteoblasts (bone-forming cells), are crucial to bone remodeling, a dynamic process where old bone is resorbed and replaced by new bone tissue. In a bone environment, bone remodeling is important to maintain appropriate bone density and BMAT. Findings of recent studies indicate that the imbalance between osteoclast activity and BMAT accumulation could result in bone-related diseases such as osteoporosis, osteoarthritis, and obesity (Shi et al., 2022; Russo et al., 2023; Rinotas et al., 2024). For instance, increased BMAT has been observed in conditions like aging, obesity, and diabetes, where excessive fat accumulation within the bone marrow contributes to bone fragility. Osteoclasts play a key role in this balance by resorbing bone tissue, which in turn affects BMAT.

Adipogenesis can also influence osteoclast differentiation (Muruganandan et al., 2020). RANKL-RANK signaling regulates the balance between osteogenesis and osteoclastogenesis, and BMADs are one of the primary sources of RANKL. Adipocytes can regulate osteoclast differentiation directly in the absence of osteogenic lineages (Goto et al., 2011). Similar to the network of co-involved transcription factors for osteogenesis and adipogenesis, transcription factors involved in adipogenesis can regulate osteoclast differentiation. PPAR-γ is a major transcription factor in the adipogenesis process and can effectively regulate obesity-related phenotypes. However, PPARγ promotes osteoclast progenitor cells by activating GATA2 transcription and regulating c-fos expression directly to promote osteoclast differentiation. In addition, PPARγ downregulates β-catenin protein and inhibits c-jun activation of PGC1β promoter expression, PGC1β also cooperates with ERRα to mediate mitochondrial biosynthesis and fatty acid oxidation, thereby activating osteoclasts (Wei et al., 2011; Wan et al., 2007a; Wei et al., 2010). Thus, PPAR-γ and its ligands may play some role in promoting osteoclast differentiation and bone resorption (Wan et al., 2007b). In addition, PPAR-γ and its agonists can regulate the level of autophagy in cells (Faghfouri et al., 2021). C/EBPα is also highly expressed in pre-osteoclasts and osteoclasts, thus promoting osteoclastogenesis by upregulating the RANK cytoplasmic IVVY motif to regulate osteoclast markers positively and negatively regulate RBP-J (Chen et al., 2013; Jules et al., 2018). Another transcription factor C/EBPβ function in adipogenesis is considered a switch for osteoclast differentiation, located downstream of the mammalian target of rapamycin kinase (mTOR), which regulates osteoclast formation by controlling the C/EBPβ isoform ratio (Smink et al., 2009; Smink and Leutz, 2010).

Studies elucidate that autophagy-related proteins, such as ATG5, ATG7, and LC3, are essential for osteoclastogenesis (DeSelm et al., 2011). Mice with deficient autophagy in osteoclasts exhibit impaired differentiation and reduced bone resorption, indicating the importance of this process for proper osteoclast activity (Aoki et al., 2020; Gong et al., 2022). Bone resorption mediated by osteoclasts involves the degradation of bone matrix proteins and the production of enzymes, such as cathepsin K, degrading mineralized bone tissue (Antika et al., 2016). Autophagy plays a key role here by maintaining the energy status of osteoclasts and removing dysfunctional components (Zhang et al., 2020a). Additionally, autophagy assists in the recycling of cellular components necessary for bone resorption, such as acidic vesicles and lysosomes (Wang et al., 2017b; Li M. et al., 2022d).

## 5 Treatment strategies related to autophagy and bone–fat balance

### 5.1 Treatment interventions related to BMMSC differentiation

The differentiation of BMMSCs under autophagy regulation significantly influences the progression of osteoporosis. Increased autophagic activity promotes osteogenic differentiation of BMMSCs while inhibiting adipogenic differentiation, thereby maintaining bone-fat balance. Modulating key signaling pathways has emerged as a research focus, with the potential to improve BMMSC osteogenic capacity (Table 2).

**TABLE 2:** Pharmacological agents targeting autophagy regulate BMMSC differentiation through a cellular signaling pathway.

Inhibitor/regulator	Pathway	Autophagy	Differentiation lineage	Treating diseases	Reference
Rapamycin	mTOR	↑	Osteogenesis↑	Osteoporosis	Li et al. (2022e)
		↑	Osteogenesis↑	Rheumatoid arthritis	Chen et al. (2022b)
Leonurine	PI3K/Akt/mTOR	↑	Osteogenesis↑	Osteoporosis	Zhao et al. (2021)
Naringin	PI3K/Akt/mTOR	↑	Osteogenesis↑	Osteoporosis	Ge and Zhou (2021)
Kaempferol	mTOR	↑	Osteogenesis↑	Osteoporosis	Kim et al. (2016) Zhao et al. (2019)
Irisin	AMPK	↓	Osteogenesis↑	Osteoporosis	Li et al. (2024b)
	Wnt/β-catenin	↑	Osteogenesis↑	Osteoporosis	Chen et al. (2020b) Colucci et al. (2021)
Ginsenoside Rg3	AMPK/mTOR	↑	Osteogenesis↑	Osteoporosis	Zhang et al. (2020b)
Alpha-lipoic acid	AMPK/mTOR	↓	Adipogenesis↓	Osteoporosis	Hahm et al. (2014) Polat et al. (2013)

### 5.2 Treatment for oxidative stress

Oxidative stress is a major pathological factor contributing to osteoporosis and bone loss. Autophagy helps maintain bone health by clearing damaged mitochondria and proteins, reducing oxidative stress accumulation, and preserving osteoblast function. Patients with osteoporosis often exhibit elevated oxidative stress levels. Mitophagy, the autophagic degradation of damaged mitochondria, alleviates oxidative damage to bone cells. Studies indicate that combining antioxidants, such as N-acetylcysteine, can significantly alleviate oxidative stress-related damage in osteoporosis (Yang et al., 2021; Abu-Kheit et al., 2022). Pharmacological activation of autophagy using agents like rapamycin and metformin may reduce oxidative stress and restore the bone-fat balance (Feng et al., 2023; Chen et al., 2023; Yang et al., 2022). Biomarkers such as LC3-II levels and mitochondrial ROS can help monitor autophagy and oxidative stress in bone metabolic disorders, including osteoporosis (Trojani et al., 2024). Stimulating autophagy offers a promising strategy to counteract oxidative stress-induced bone loss and fat accumulation, particularly in aging and metabolic diseases (Tong et al., 2022; Huang et al., 2022; Shi et al., 2020; Wei et al., 2023).

Glucocorticoid-induced oxidative stress and mitochondrial damage are key contributors to secondary osteoporosis. However, GCs also induce oxidative stress and indicate a biphasic property; the dose of GCs determines the fate of osteoblasts toward autophagy or apoptosis (Wang et al., 2020a; Jia et al., 2011). High-dose DEX (≥10^−6^ M) constantly inhibits osteoblast viability and induces cell apoptosis, whereas low-dose DEX (10^−8^ M)increases cell viability (Zhang et al., 2018b). This DEX-induced cell apoptosis could result from the mitochondria fusion under increasing oxidative stress (Hsu et al., 2020). Summarizingly, activated autophagy is a self-protective mechanism of osteoblasts, resistance to the stress induced by excess GCs. These studies elucidate that autophagy responds to oxidative stress and regulates bone-fat balance in a secreted way. Autophagy mitigates these effects by clearing damaged mitochondria, reducing osteoblast apoptosis, and enhancing therapeutic outcomes.

### 5.3 Treatment for aging

Autophagic activity declines with aging, leading to the accumulation of damaged proteins and mitochondria, increased oxidative stress, and impaired bone health. Enhancing autophagy has been shown to mitigate age-related bone loss, reduce bone marrow fat accumulation, and improve bone-fat balance, thus highlighting its potential as a therapeutic target for anti-aging and bone health maintenance.

Polyphenol compounds (such as resveratrol and curcumin) activate autophagy-related signaling pathways, reduce aging-related bone loss, and maintain bone-fat balance (Li et al., 2024a; Qin et al., 2017; Wang et al., 2020b). Enhancing autophagy through genetic interventions may prevent senescence, promoting osteogenesis and reducing BMAT accumulation (Lian et al., 2021; Huang et al., 2021). Autophagy activation could provide therapeutic benefits for conditions like osteoporosis and obesity-related bone loss by rebalancing the bone-fat balance. The modulation of senescence through autophagy offers new strategies for treating age-related musculoskeletal disorders, with implications for regenerative medicine and anti-aging therapies.

### 5.4 Treatment for inflammatory bone disease

Chronic inflammatory conditions, such as rheumatoid arthritis and ankylosing spondylitis, disrupt the bone-fat balance through pro-inflammatory cytokines like TNF-α and IL-1β, resulting in greater bone resorption and fat deposition (Taylan et al., 2012; Schett and Gravallese, 2012). These processes accelerate the progression of osteoporosis and bone marrow adiposity. Autophagy plays a critical role in regulating immune responses and inflammation, thereby offering a means to alleviate inflammation, protect bone tissue, and prevent excessive fat accumulation. This makes autophagy modulation a promising approach for treating inflammatory bone diseases.

Inflammation-induced oxidative stress and metabolic dysregulation significantly impair the differentiation of BMMSCs. Autophagy addresses these challenges by clearing oxidative stress products and inflammatory mediators, thus promoting osteogenic differentiation while suppressing adipogenesis, and preserving the metabolic health of a bone (Zhang et al., 2024; Li et al., 2023; Wang et al., 2022b).

Furthermore, inflammation-driven bone marrow adiposity not only undermines skeletal health but is also associated with systemic metabolic disorders, such as insulin resistance (Guimarães et al., 2024). Activating autophagy to regulate the bone-fat balance may indirectly improve systemic metabolic health, providing broad therapeutic potential. By inhibiting the release of inflammatory mediators (e.g., suppressing the NLRP3 inflammasome) and modulating immune cell functions (e.g., macrophage polarization), autophagy offers a multifunctional strategy to protect bone tissue, reduce fat deposition, and address inflammation in the treatment of inflammatory diseases (Hu et al., 2023; Cheng et al., 2024).

## 6 Outlook

In conclusion, the balance between bone and BMAT mediates the crosstalk between bone and lipid metabolism. Contrastively, based on the triad of interactions among osteoblasts, adipocytes, and osteoclasts at the cellular level, enhanced bone marrow adipogenesis leads to decreased osteogenic differentiation of BMMSCs and promotes osteoclast formation to accelerate bone loss. Furthermore, obesity and lipid metabolism affect bone, and bone can regulate BMAT function and energy metabolism as an endocrine organ. Autophagy is a cellular mechanism that primarily serves to protect itself through the eat-me signal. Autophagy is also associated with lipid metabolism for its responsiveness to nutrients. Autophagy drives lipolysis and maintains lipid homeostasis by regulating lipid signaling pathways. It can also remove accumulated free lipid metabolic products or inflammation to ensure a relatively stable level of oxidative stress in skeletal cells. However, unbalanced autophagy homeostasis can also lead to bone diseases. Thus it can be proposed that autophagy can link and balance bone-lipid metabolism to affect bone health. Much uncertainty still exists about the topic. We still need to understand this process more comprehensively and identify the signaling pathways and molecules that play a role in autophagy in bone-fat homeostasis. Autophagy is a novel field of drug development and a potential therapeutic approach for bone metabolic diseases by affecting bone-fat metabolism through autophagy.

## Glossary

AMPK: AMP-activated protein kinase
ATG: autophagy-related gene
ATM: adipose tissue macrophage
ATP: adenosine triphosphate
BMAD: bone marrow adipocyte
BMAT: bone marrow adipose tissue
BMMSC: bone marrow mesenchymal stem cell
BMP: bone morphogenetic protein
DEX: dexamethasone
ECM: extracellular matrix
EPA: eicosapentaenoic acid
ER: endoplasmic reticulum
EPO: erythropoietin
GC: glucocorticoid
HFD: high-fat diet
HO-1: heme oxygenase-1
IL: interleukin
LD: lipid droplet
LPS: lipopolysaccharide
MMP: matrix metalloproteinase
MSC: mesenchymal stem cell
mTORC1: mechanistic target of rapamycin kinase complex 1
mTORC2: mechanistic target of rapamycin kinase complex 2
OA: osteoarthritis
OCN: osteocalcin
OPG: osteoprotegerin
OPN: osteopontin
PA: palmitate
PPARγ: peroxisome proliferator‐activated receptor-γ
PTH: parathyroid hormone
PUFA: polyunsaturated fatty acid
ROS: reactive oxygen species
SASP: senescence-associated secretory phenotype
SOST: sclerostin
TNF: tumor necrosis factor
